# Wilson’s disease presenting with HELLP syndrome; A case report

**DOI:** 10.4274/tjod.24434

**Published:** 2015-03-15

**Authors:** Sümeyra Nergiz Avcıoğlu, Sündüz Özlem Altınkaya, Mert Küçük, Emre Zafer, Selda Demircan Sezer, Ali Rıza Odabaşı

**Affiliations:** 1 Adnan Menderes University Faculty of Medicine, Department of Gynecology and Obstetrics, Aydın, Turkey; 2 Muğla Sıtkı Koçman University Faculty of Medicine, Department of Gynecology and Obstetrics, Muğla, Turkey; 3 Private Outpatient Clinic, Aydın, Turkey

**Keywords:** Wilson’s disease, Pregnancy, HELLP syndrome, acute liver failure

## Abstract

Wilson’s disease (WD) is an autosomal recessive disorder. It is characterized by toxic accumulation of copper mainly in the liver and brain but also in cornea and kidney due to a defect in biliary excretion of copper. The hepatic manifestation of WD is diverse and may include asymptomatic elevation of aminotransferase, chronic hepatitis, cirrhosis, or acute/fulminant hepatic failure. Characteristic of acute hepatic failure in WD is concomitance of acute intravascular hemolytic anemia. Acute intravascular hemolytic anemia and thrombocytopenia in WD may be interpreted as a feature of Hemolysis, Elevated Liver Enzymes, Low Platelet Count (HELLP) syndrome besides acute liver failure. The differential diagnosis may be very difficult. Here, WD in pregnancy presenting with clinical symptoms of HELLP syndrome and developing acute liver failure in postpartum period is discussed.

## INTRODUCTION

Wilson’s disease (WD) was first identified by Kinnear Wilson in 1912. The incidence of the disease is reported as 1/30000-1/100000 births^(1)^. Carrier frequency is 1/90. The gene responsible for the disease is ATP7B, located on chromosome 13^(2)^. It is most commonly seen in the second-third decades of life^(3)^. In more than 80% of patients, the symptoms of the disease are observed in the first three decades^(4)^. The symptoms of WD-related fulminant hepatic failure are particularly seen in female patients below the age of 40 years^(5)^. Clinical findings are quite various and include hepatic and neuropsychiatric symptoms. The hepatic manifestation of WD is diverse and may include a clinical presentation that varies from asymptomatic elevation of aminotransferase, chronic hepatitis and cirrhosis, to acute/fulminant hepatic failure^(6)^.

The acute liver failure in WD is concomitant with acute intravascular Coombs-negative hemolytic anemia and, in some patients, may be manifested as the first symptom of the disease^(7)^. Acute intravascular hemolysis and thrombocytopenia in a pregnant woman known to have WD may be interpreted, together with acute liver failure, as a feature of HELLP syndrome^(8)^. The differential diagnosis may be difficult however. HELLP syndrome is seen in 0.1-0.6% of pregnant women, particularly in the second trimester (in 70% of cases) and in the postpartum period (in the first 1-2 days)^(9)^. The laboratory criteria for HELLP syndrome were first defined in 1986. These are hemolytic findings (bilirubin increase >1.2 mg/dL), abnormal liver functions (AST>70 IU/L), reduced thrombocytes of less than 100x109/L. Endothelial damage or dysfunction causes abnormal expression of inflammatory mediators and an activation and aggregation of thrombocytes. This leads to the fragmentation of red blood cells, hemolysis and microangiopathic anemia. In addition, an abnormal activation of thrombocytes and thrombocytopenia arising from increased use are observed^(4)^. Here, a case of WD in pregnancy, presenting clinical manifestations of HELLP syndrome concomitant with acute liver failure developed during postpartum period is discussed.

## CASE

**Figure 1:**
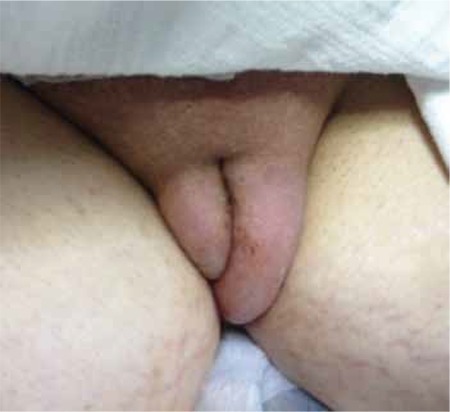
Vulvar edema due to acute liver failure in Wilson’s disease (WD)

A 24-year-old patient in the 37^th^ week of spontaneous pregnancy, gravida 1, parity 0, was admitted at our obstetrics clinic with the start of labor. The patient’s pregnancy controls were taking place at our obstetrics outpatient clinic as from the 31^st^ week of pregnancy. An abdominal ultrasonography performed when the patient was seventeen years old revealed a cirrhotic liver. The biochemical test results showed a ceruloplasmin level of 10.1mg/dl (25.0-45.0 mg/dl), free copper levels of 71μgr/dl (normal <15 μgr/dl), a urine copper level of 120 μgr/24 hr (normal: <100 μgr/24 hrs), upon which a liver biopsy was performed leading to a diagnosis of Wilson’s disease. From an investigation into the patient’s family history, it was learned that a sibling had undergone a liver transplant due to Wilson’s disease. Also followed up by the gastroenterology outpatient clinic, the patient was being treated with 300 mg 3x1 of Metalcaptase (D-penicillamine) and 2x1 p.o. of Zinco (50 mg zinc) throughout her pregnancy but she stopped taking this treatment for the last three days. On the patient’s examination, blood pressure was 140/90 mmHg and in complete urinalysis, proteinuria was found to be at +++ level. Furthermore, the following results were obtained in the blood tests; AST: 45 U/L (5-40 U/L), ALT: 20 U/L (5-40 U/L), ALP: 216 U/L (40-150 U/L), LDH: 406 U/L (125-243 U/L), amylase: 47 U/L, total bilirubin: 1.2 mg/dL (0.2-1.2 mg/dL), direct bilirubin: 0.64 mg/dL (0-0.5 mg/dL), total protein: 4.7 g/dL (6.4-8.3 g/dL), albumin: 2 g/dL (3.5-5 g/dL), thrombocytes: 98x10^9^/L (150-300x10^9^/L). The patient was diagnosed with pre-eclampsia. The additional pelvic examination revealed pronounced cephalopelvic disproportion and a cesarean section was therefore carried out. The patient delivered a live 3050-gram male infant in cephalic presentation with an APGAR score of 7-9. The patient was followed up on the hospital ward in the postpartum period. A change in the laboratory tests was observed at 24 hours postoperatively. The clinical results were as follows: AST: 50 U/L (5-40 U/L), ALT: 19 U/L (5-40 U/L), total protein: 4.1 g/dL (6.4-8.3 g/dL), albumin: 1.6 g/dL (3.5-5 g/dl) and thrombocyte values: 89x10^9^/L (150-300x10^9^/L). A gastroenterological evaluation of the patient was ordered but the patient was discharged on the 2^nd^ day postoperatively on her own request. On the third postoperative day, the patient was admitted to the emergency room with the complaints of widespread swelling in the abdomen, vulva and legs. Physical examination revealed massive vulvar edema, pretibial edema in the lower extremities, and the abdomen was distended (Figure 1). Blood pressure was 140/90 mmHg, pulse rate was 87/min and temperature 36.8 ºC. Ultrasonography showed an uterus of postpartum size with widespread free fluid in the abdomen. The test results were as follows; Hb (hemoglobin): 10.2 g/L (11.7-15.5 gr/dL), hematocrit: 28.4% (37-44%), thrombocytes: 64x109/L (150-300x109/L), CRP: 58.99 mg/L (0-6 mg/L), AST: 89 U/L (5-40 U/L), ALT: 30 U/L (5-40U/L), ALP: 132 U/L (40-150 U/L), LDH: 305 U/L (125-243 U/L), total protein: 3.7 g/dL (6,4-8,3 g/dL), albumin: 1.5 g/dL (3.5-5 g/dL), total bilirubin: 3.0 mg/dL (0.2-1.2 mg/dL), direct bilirubin: 1.55 mg/dL (0-0.5 mg/dL). Widespread pulmonary infiltration was observed in posteroanterior direct X-ray. After an internal medicine examination of the patient, human albumin replacement and treatment with a diuretic was planned. The patient was kept under close monitoring and control. After the consultation of gastroenterology department, administration of metalceptase (D-penicillamine) 2x1, aldactone (spironolactone 100 mg) 1x1, desal (furosemide 40 mg) 1x1 were recommended. The treatment, which is ongoing, resulted in slight regression in symptoms of liver failure such as vulvar edema, abdominal ascites, pulmonary edema and shortness of breath. The diagnosis given by gastroenterology department was WD-related acute liver failure. Laboratory tests showed AST: 87 U/L (5-40 U/L), ALT: 39 U/L (5-40 U/L) and LDH: 393 U/L (125-243 U/L), albumin: 2.5 g/dL (3.5-5 g/dL). Hematological tests showed leukopenia (WBC: 2.07x10^9^/L, normal: 4.1-10.9x10^9^/L) and a drop in blood values (Hb): 10.7 g/L (11,7-15.5 gr/dL), hematocrit (Hct): 30.1% (37.0-44%), RBC: 3.30x106/mcrL (3.83-5.08 106/mcrL), PLT: 40x10^9^/L (150-300x10^9^/L). The values in the coagulation profile were; prothrombin time (INR-international normalized ratio): 1.29 (08-1.2), Aptt: 41.1s (26.6-40.0), fibrinogen: 142 (180-350), D-dimer: 4871 (50-228). Kidney function tests were normal. When a full recovery could not be seen in the clinical and laboratory findings with the ongoing treatment and because of the need for a liver transplantation in the long term, the patient was referred to a hospital that had an organ transplantation department. The patient has been waiting her turn for a liver transplantation for the last two months and the current treatment continues, (the consent of the patient has been obtained, verbally and in written form, for the presentation of this case study).

## DISCUSSION

WD is an autosomal recessive disease. It is characterized by toxic accumulation of copper, mainly in the liver and brain but also in the cornea and kidney, due to a defect in the biliary excretion of copper^(3)^. The symptoms of WD comprise hepatic findings at a rate of 40-70%^(6,10)^ and neuropsychiatric findings at a rate of 50%^(11)^. Hepatic symptoms are more pronounced in children while neurological and psychiatric symptoms are more common in adults^(12)^. In this case however the patient presented with hepatic symptoms and no neuropsychiatric symptoms were observed.

Neurological and psychiatric adversities in patients with WD preclude the possibility of marriage^(13)^. Furthermore, it is not uncommon for a woman in this category to be suffering from amenorrhea, oligomenorrhea, irregular menstruation, and repeated miscarriages. In symptomatic cases, hormonal changes related to liver dysfunction disrupt fertility. The diffusion of non-ceruloplasmin-bound copper from plasma into tissues may affect ovarian follicular aromatase activity^(14)^. Earlier studies have reported healthy full-term pregnancies in treated patients^(15)^, but miscarriage and pregnancy loss rates show an increase in untreated cases^(16)^. Here, we report a case with spontaneous pregnancy at full term with no previous treatment for infertility.

The aim of treatment is the removal of copper from the body and the prevention of copper absorption through diet^(13)^. D-penicillamine increases excretions of copper in the urine by creating non-toxic copper-penicillamine compounds; it is the first preference for treatment^(1,2)^. Patients who do not tolerate this treatment are given trientine as an alternative. Zinc competes with copper and prevents intestinal copper absorption, proving to be an effective medication in long-term maintenance of the disease^(13)^. In the present case, the treatment option of penicillamine (metalcaptase) was chosen.

D-penicillamine is recommended for treatment during pregnancy since it is well-tolerated and safe for both mother and child. A smaller dose is used in the treatment of WD and it is rapidly eliminated from the body after binding with copper by chelation and its level decreases even lower. It is because of this reason, treatment with penicillamine during pregnancy has been reported to be safe^(17)^. In addition, treatment should continue in pregnancy in order to avert the development of hemolysis, liver dysfunctions and liver failure^(18)^. Dosage should be kept at the lowest effective level and the dose should be reduced in last trimester to 25-50% of the dose administered before the pregnancy^(19)^ in order to avoid interfering with wound-healing. We believe that in the present case, the patient was treated with penicillamine throughout the entire pregnancy and stopping the drug in the last three days triggered the acute liver failure. There is no other publication in the literature reporting development of acute liver decompensation in such a short time after the cessation of treatment. In fact, Shimono et al.^(20)^ reported a case of pregnancy with no problems during treatment with D-penicilline which yielded two births. Treatment was stopped at the third child of the same patient and acute liver failure caused by hemolytic crisis stemming from the accumulation of copper in the body occurred after the delivery. We also believe that acute liver failure seen in the postpartum period in the present case was caused by the same mechanism.

In this report, a case of WD in pregnancy, presenting with the clinical manifestations of HELLP syndrome concomitant with postpartum acute liver failure has been discussed. Since no microscopic blood analysis was performed, a direct microscopic imaging of hemolytic anemia was not carried out. At first presentation with HELLP syndrome, total bilirubin was 1.2 mg/dl and direct bilirubin was 0.64 mg/dl; in the period when the liver failure developed, total bilirubin was found to be 3.00 mg/dl. As it was in this case, even though total bilirubin is normal or minimally increased (1-2 mg/dL) in HELLP syndrome^(21)^, this value would have been much higher in acute liver failure(22). At the same time, this patient had leukocytosis, an elevated D-dimer and reduced serum albumin. Although these findings are less typical in HELLP syndrome,^(21,23)^ the proteinuria found in the spot urine test (+++) does suggest HELLP^(17)^. Elevations in serum aminotransferase in WD are less than 10-fold, as in this case^(22)^. Moreover, there were also some anomalies found in the coagulation test; there were prolongation of INR and APTT values. In addition, the fact that blood pressure measured as 140/90 mmHg indicates WD together with HELLP syndrome. The normal kidney function tests of the patient steered us away from hepatorenal syndrome. On the other hand, the patient progressed into acute liver failure in the postpartum period.

To conclude, the main objective in following up cases of concomitant WD and pregnancy is to evaluate maternal and fetal effects and to control the disease using the lowest possible dosage of drug. A multidisciplinary approach and the close monitoring of high-risk pregnancies will lead to similar positive outcomes in the general population. WD is a chronic liver disease that allows a normal life if diagnosed and treated early. It is a condition that responds to treatment, even when cirrhosis has been diagnosed, but needs life-long treatment and care. Treatment should continue over the life span and should only be stopped when a liver transplant has been performed. Even a short interruption in the treatment of only three days in this case has the potential of speeding up the process of acute hepatic failure and the development of decompensation.
